# Exhaled Aerosol Pattern Discloses Lung Structural Abnormality: A Sensitivity Study Using Computational Modeling and Fractal Analysis

**DOI:** 10.1371/journal.pone.0104682

**Published:** 2014-08-08

**Authors:** Jinxiang Xi, Xiuhua A. Si, JongWon Kim, Edward Mckee, En-Bing Lin

**Affiliations:** 1 School of Engineering and Technology, Central Michigan University, Mount Pleasant, Michigan, United States of America; 2 Science Division, Calvin College, Grand Rapids, Michigan, United States of America; 3 College of Medicine, Central Michigan University, Mount Pleasant, Michigan, United States of America; 4 Department of Mathematics, Central Michigan University, Mount Pleasant, Michigan, United States of America; University of Jaén, Spain

## Abstract

**Background:**

Exhaled aerosol patterns, also called aerosol fingerprints, provide clues to the health of the lung and can be used to detect disease-modified airway structures. The key is how to decode the exhaled aerosol fingerprints and retrieve the lung structural information for a non-invasive identification of respiratory diseases.

**Objective and Methods:**

In this study, a CFD-fractal analysis method was developed to quantify exhaled aerosol fingerprints and applied it to one benign and three malign conditions: a tracheal carina tumor, a bronchial tumor, and asthma. Respirations of tracer aerosols of 1 µm at a flow rate of 30 L/min were simulated, with exhaled distributions recorded at the mouth. Large eddy simulations and a Lagrangian tracking approach were used to simulate respiratory airflows and aerosol dynamics. Aerosol morphometric measures such as concentration disparity, spatial distributions, and fractal analysis were applied to distinguish various exhaled aerosol patterns.

**Findings:**

Utilizing physiology-based modeling, we demonstrated substantial differences in exhaled aerosol distributions among normal and pathological airways, which were suggestive of the disease location and extent. With fractal analysis, we also demonstrated that exhaled aerosol patterns exhibited fractal behavior in both the entire image and selected regions of interest. Each exhaled aerosol fingerprint exhibited distinct pattern parameters such as spatial probability, fractal dimension, lacunarity, and multifractal spectrum. Furthermore, a correlation of the diseased location and exhaled aerosol spatial distribution was established for asthma.

**Conclusion:**

Aerosol-fingerprint-based breath tests disclose clues about the site and severity of lung diseases and appear to be sensitive enough to be a practical tool for diagnosis and prognosis of respiratory diseases with structural abnormalities.

## Introduction

Accurate and early diagnosis of lung cancer is crucial to patients’ survivability. For instance, patients with non-small cell lung cancer have a cure rate of more than 70% when diagnosed at Stage I whereas less than 25% if diagnosed at Stage III [1]. Conventional methods of diagnosing lung diseases or cancers include pulmonary function tests using the chest X-ray for screening, CT/PET/SPET for examining abnormal structures, and sputum cytology or lung tissue biopsy for evaluating the type and extent of the cancer [2]. These diagnosis procedures are generally reliable, but are costly and require professional operations. Moreover, some procedures are invasive and pose radiation risks to patients. Recently, an alternative diagnosis method using a patient’s exhaled breath has been developed based on the premise that exhalation contains clues to many diseases [3]. Metabolic changes of growing cancer cells cause changes in the production of certain chemicals and generate a unique breath “*fingerprint*”, which can be used to determine whether a disease is present. Studies have reported elevated levels of nitric oxide in relation with asthma [4], antioxidants with chronic obstructive pulmonary disease (COPD) [5], chemokines with cystic fibrosis [6], and isoprene with non-small cell lung cancer (NSCLC) [7]. Reviews on evidence supporting lung cancer diagnosis using breath tests and related developments of breath devices can be found in [8], [9]. These breath devices are often small in size, noninvasive, easy to use, less expensive, and hold the promise of efficient diagnosis of lung cancer and other respiratory diseases.

**Figure 1:**
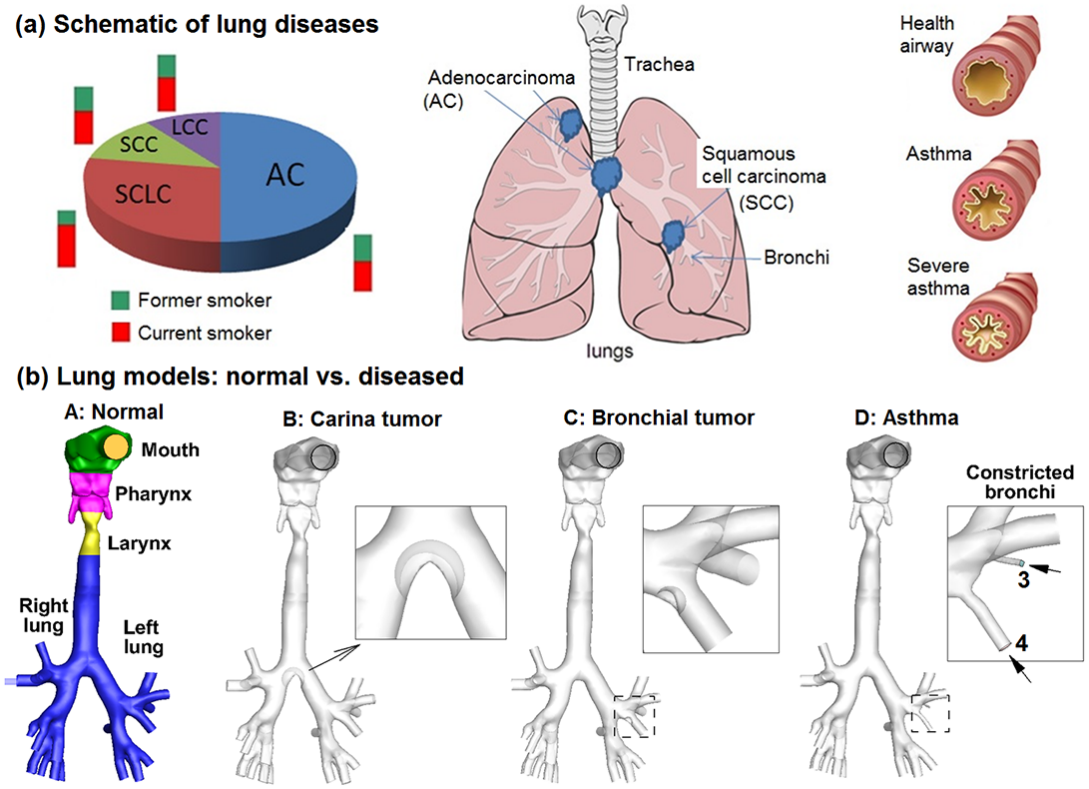
Schematic of lung diseases and airflow dynamics. (**a**) Lung diseases subtypes: squamous cell cancer *(SCC)*, adenocarcinoma (AC), large cell cancer (LCC), and small cell lung cancer (SCLC), and asthma. (**b**) Lung models with healthy and diseased conditions: Model A with normal airway structure, Model B with an adenocarcinoma at the carina ridge (carina tumor), Model C with a squamous cell carcinoma on a left segmental bronchus (bronchial tumor), and Model D with constricted segmental bronchi (asthma).

In spite of these advantages, gas-signature based breath devices only measure the presence and concentration of exhaled gas chemicals. They do not provide information on where these chemicals are produced (the cancer site) or the level of airway remodeling, both of which are crucial in cancer treatment planning. The site and degree of airway remodeling can be substantially different for different lung cancers (Fig. 1a). Any alternative that can locate the malignant sites in a safer and less expensive way would be highly desirable. Currently, this information can only be obtained with the help of radiological techniques such as CT or PET. A number of studies have explored the use of aerosols as a lung diagnostic tool, such as the aerosol bolus dispersion (ABD) method [10], [11], [12]. However, the ABD method does not provide new information about the lung function compared to existing pulmonary function tests [12]. More recently, Xi et al. [13] proposed a new aerosol breath test that has the potential to detect the disease and locate its site. This method arises from persistent observations of unique deposition patterns with respect to prescribed geometry and breathing conditions [14], [15], [16]. We hypothesize that each airway structure has a signature *aerosol fingerprint* (AFP), as opposed to the *gas fingerprint* discussed before. Accordingly, any deviation from the normal pattern may indicate an abnormality inside the airway, which can be retrieved with an inverse numerical approach developed by Xi et al. [17]. The subsequent questions are: how can we quantitate the exhaled AFP patterns from different airway geometries? Will the exhaled AFPs be sensitive enough to detect airway structural changes? More importantly, how can we use this information to predict the presence and location of airway abnormalities based on samples of exhaled aerosol profiles?

In this study, fractal analysis will be implemented to quantitate the complex patterns of exhaled aerosol fingerprints. These patterns, although visually distinguishable, are resistant to automatic quantitation and comparison. Since its introduction by Mandelbrot [18], fractal analysis has been shown to be a robust and powerful tool to measure the subtle changes in biological morphology [19], vasculature [20], neural networks [21], metal structures [22], [23], landscapes [24], and even the stock market [25]. Fractal geometry provides a simple model to describe complex systems with a minimum number of parameters (e.g., fractal dimension specifying the degree of irregularity or complexity). The conducting airways of human lungs are “space filling” fractal structures [26], [27]. Studies of the bronchial tree have shown that the mean diameter of the airways is exponentially related to the order of branching [28] with a fractal dimension of 1.57 [29]. Considering that tracer particles sequentially fill and empty the fractal lung during inhalation and exhalation, it is conceivable that the exhaled aerosol profiles also exhibit fractal characteristics and are thus amenable to fractal analysis.

However, fractal analysis has two inherent limitations. First, fractal dimension describes the complexity of an image by quantifying *how much* space is filled by the particles; however, it does not explain how the space is filled by these particles. To address this limitation, lacunarity is also evaluated to describe the spatial pattern of exhaled aerosol fingerprints. Lacunarity is a measure of heterogeneity that describes the distribution of empty spaces surrounding the particles. Patterns with high lacunarity are more heterogeneous while those with low lacunarity are more homogenous or rotationally invariant. Lacunarity adds significantly to the description of an image with a known fractal dimension, in that it describes the empty spaces in the image, and thus describes how the particles fill the space. Therefore, lacunarity can be used to differentiate aerosol patterns with similar fractal dimensions, which may fill the space differently. The second limitation of simple fractal analysis is that one single fractal dimension alone may not adequately describe the complex patterns of exhaled aerosol fingerprints, which consist of different scales or details [13]. Considering that airflows within the lungs result from a multiplicative cascade of non-linear processes [30], even small variations in the lung morphology could appreciably alter the exhaled aerosol patterns. Compared to monofractal dimension analysis, multifractal analysis provides more information about the space filling properties and thus will be more appropriate to quantify the exhaled aerosol profiles. Reviews of monofractals and multifractals can be found in [31].

The objective of this study is to assess the feasibility of aerosol breath tests in diagnosing the location and severity of obstructive lung diseases. We will first evaluate the sensitivity of exhaled AFPs to airway modifications by computationally testing four lung models (one benign and three malign conditions). To simulate a breath test, aerosols are first inhaled and subsequently exhaled, with exit aerosol profiles being captured at the mouth. The exhaled AFPs will then be quantified using fractal and lacunarity analysis to yield a more compact and simplified representation of the particle spatial distributions. This will help to better correlate the aerosol patterns and airway diseases. An automated pipeline of pattern characterization and classification can also expedite processing of large amounts of images in the future.

## Methods

### 2.1. Construction of airway models with normal and malign conditions

To evaluate the sensitivity of exhaled aerosol profiles to morphology variations of the upper respiratory airway, four models were considered in this study. The first one, Model A, extended from the mouth to the bronchial bifurcations G6 and was originally developed by Xi and Longest [14] based on MRI images of a healthy adult male. Details of the airway geometry, including the construction procedures and critical dimensions, could be found in Xi and Longest [14], [32] and were described briefly as follows. The multi-slice MRI scans of the subject were segmented using MIMICS (Materialise, Ann Arbor, MI) into a 3-D model, which was further converted into a set of contours that defined the airways of interest. Based on these contours, an internal surface geometry was constructed in Gambit. Surface smoothing was performed to the least extent to preserve the airway anatomical details as much as possible. The resulting model was intended to represent a normal airway and was further modified to generate the other three models with different abnormalities in the tracheobronchial (TB) region, as shown in Fig. 1b. Model B had a 10 mm tumor located at the tracheal carina ridge. Model C had a smaller sized tumor (4 mm) at the segmental bronchi in the left lower lobe. The tumor-to-airway diameter ratios selected here were consistent with those adopted by Segal et al. [33], who studied the impact of tumor size and locations in TB airways. Model D had two severely constricted segmental bronchi in the left upper lobe (number 3 and 4 in Fig. 1b), and represented asthmatic airways. Morphologically, Model B represents a large airway obstruction, Model C a small airway obstruction, and Model D a severe flow perturbation. The detailed information of location and size of the four models was listed in Table 1.

**Table 1:** The location and size of airway models with benign and malign conditions.

Reference	Condition	Location	Size (mm)
Model A	Normal	*LSB (3^rd^)	8.0
		LSB (4^th^)	8.4
Model B	Large tumor	Tracheal carina	10
	(35% blockage)		
Model C	Small tumor	LSB (4^th^)	4.0
	(47% blockage)		
Model D	Asthma		
	75% constricted	LSB (3^rd^)	2
	53% constricted	LSB (4^th^)	4

*LSB: Left segmental bronchus.

### 2.2. Numerical breath test protocol

There were two steps in this protocol: image acquisition via computational modeling and image analysis via fractal and lacunarity analysis. In light of computational modeling to acquire exhaled aerosol fingerprints (AFPs), both inhalation and exhalation were simulated in this study, with a bolus of tracer particles first inhaled slowly and then exhaled. It is assumed that inhaled ambient air or mainstream smoke will enter the mouth-throat (MT) geometry with a relatively blunt velocity profile, which can be defined as
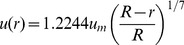
(1)where *r* is the inlet radial coordinate, *u_m_* is the mean velocity and *R* is the radius of the inlet. This profile is similar to a constant velocity inlet, but provides a smooth transition to the no-slip wall condition. A stochastic model was used to generate the inlet particle profiles with initial particle velocities matching the local fluid velocities. Five particle inlet profiles were simulated for each model. During inhalation, atmospheric and vacuum pressures were assumed at the mouth and bronchial outlets, respectively. Aerosols were released at the mouth, and recorded at the outlets. During exhalation, the recorded bronchial particle profiles were specified as the inlet conditions and were tracked with expiratory airflow. The exhaled aerosols were collected at the mouth.

The exhaled particle profiles (or AFPs) were then visualized and analyzed in order to classify the AFP patterns among airway models with normal and malign conditions. The computationally predicted results were shown in the form of particle locations, particle concentration distribution, and relative concentration to the normal condition. The resultant images were then quantitated using (1) statistical distribution in translational, radial, and circumferential directions to describe the spatial pattern of the AFPs, (2) regional and localized fractal analysis, (3) lacunarity analysis, and (4) regional and localized multifractal analysis. The involved algorithms will be explained below.

### 2.3. Computational fluid-particle transport models

Flows in this study were assumed to be isothermal and incompressible. Continuous inhalation and normal breathing conditions were assumed for all simulations. A large eddy simulation approach LES-WALE model was used to solve the flow field, which included a resolved part and a sub-grid part. The resolved part of the field represented the ‘large’ eddies and were solved directly, while the sub-grid part of the velocity represented the ‘small scales’ whose effect on the resolved field was included through the sub-grid-scale (SGS) model. The LES-WALE model had been shown to produce almost no eddy-viscosity in wall-bounded laminar flows and was therefore capable of reproducing the laminar-to-turbulent transition [34]. A more detailed mathematical description of the LES-WALE model was given in Nicoud and Ducros [34].

The trajectories of monodispersed particles with a diameter (*d_p_*) were calculated on a Lagrangian basis by directly integrating an appropriate form of the particle transport equation [14],
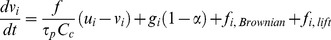
(2)where *v_i_* is the particle velocity, *u_i_* is the local fluid velocity, and *τ_p_* (i.e., *ρ_p_ d_p_* ^2^/18 *µ)* is the characteristic time required for a particle to respond to changes in the flow field. The particle residence time *τ_p_* is defined as *ρ_p_ d_p_* ^2^/18 *µ,* with *µ* being the air viscosity and *d_p_* the particle diameter. The drag factor *f* is based on the expression of Morsi and Alexander [35]. The Cunningham correction factor *C_c_* was computed using the expression of Allen and Raabe [36]. The effect of Brownian motion was considered [37] due to the small particle size in this study. In-house user-defined functions (UDFs) were implemented that considered the near-wall damping effect [32] and the finite particle inertial effect [38]. In our previous studies, the UDF-enhanced Lagrangian model had been shown to provide close agreement with experimental deposition data in upper respiratory airways for both submicrometer [39] and micrometer particles [16], [40], [41].

The computational meshes of the four airway models were generated with ANSYS ICEM CFD (Ansys, Inc). Due to the high complexity of the model geometries, unstructured tetrahedral meshes were generated with high-resolution prismatic cells in the near-wall region. A grid sensitivity analysis was conducted by testing the effects of different mesh densities with approximately 600 k, 1.2 million, 2.0 million and 3.2 million control volumes while keeping the near-wall cell height constant at 0.05 mm. Since the changes in both total and sub-regional depositions were less than 1% when increasing mesh size from 2 million to 3.2 million, the final grid for reporting flow field and deposition conditions consisted of approximately 2 million cells with a thin five-layer pentahedral grid in the near-wall region and a first near-wall cell height of 0.05 mm.

### 2.4. Fractal and lacunarity analysis

#### Box counting fractal dimension (D_B_)

**D_B_** was a measure of increasing details with decreasing resolution scales. It was calculated as the slope of the regression line of the log-log plot of box size (or scale,*ε*) and box count N_*ε*_, which is the number of grid boxes containing pixels.
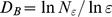
(3)


*Lacunarity (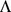):* As a measure of heterogeneity, lacunarity was calculated as
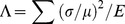
(4)where σ is the standard deviation, µ is the number of pixels per box at size ε, and E is the total number of box sizes [19], [42]. The sliding box algorithm was implemented to calculate the lacunarity 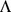 [19]. The resulting lacunarity was independent of the D_B,_ and patterns indistinguishable by their D_B_ were often distinguishable by 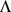, or vice versa [19].

*Multifractal spectrum f(α) ∼ α:* The multifractal analysis relies on the fact that natural systems often possess rich scaling properties. To calculate the multifractal dimensions, a normalized measure 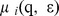 was constructed with a family of scaling exponents, *q,* to explore different regions of the singularity measure,
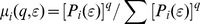
(5)For q>1, 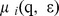 amplified the more singular measure, while for q<1, it accentuated the less singular regions. The singularity strength 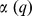
and the multifractal spectrum function f(α) with respect to 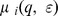
were given by



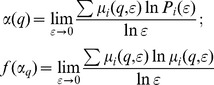
(6)The plot f(α)∼α constituted the multifractal spectrum. To calculate the lacunarity 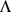 and multifractal parameters α and f(α), ImageJ with FracLac plugin was used [43].

*Generalized Fractal Dimension (D_q_):* The generalized dimension, *D_q_,* addressed how mass varied with ε and provided a direct measurement of the fractal properties of the image, which was defined as below:
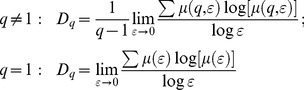
(7)


The plot of *Dq*∼*q* tended to be a decreasing sigmoidal for multifractals and horizontal for non- or monofractals.

### 2.5. Statistical analysis

Exhaled aerosol data are presented as mean ± standard deviation (SD) based on the five breath tests for each model. Data analysis was performed using the SAS statistical package (SAS Institute, Inc.). A Kruskal-Wallis one-way analysis of variance test was used to compare the difference in exhaled aerosol patterns of different models in terms of their fractal dimensions and lacunarities. A difference was considered statistically significant if p was<0.05.

## Results

### 3.1. Airflow field

**Figure 2:**
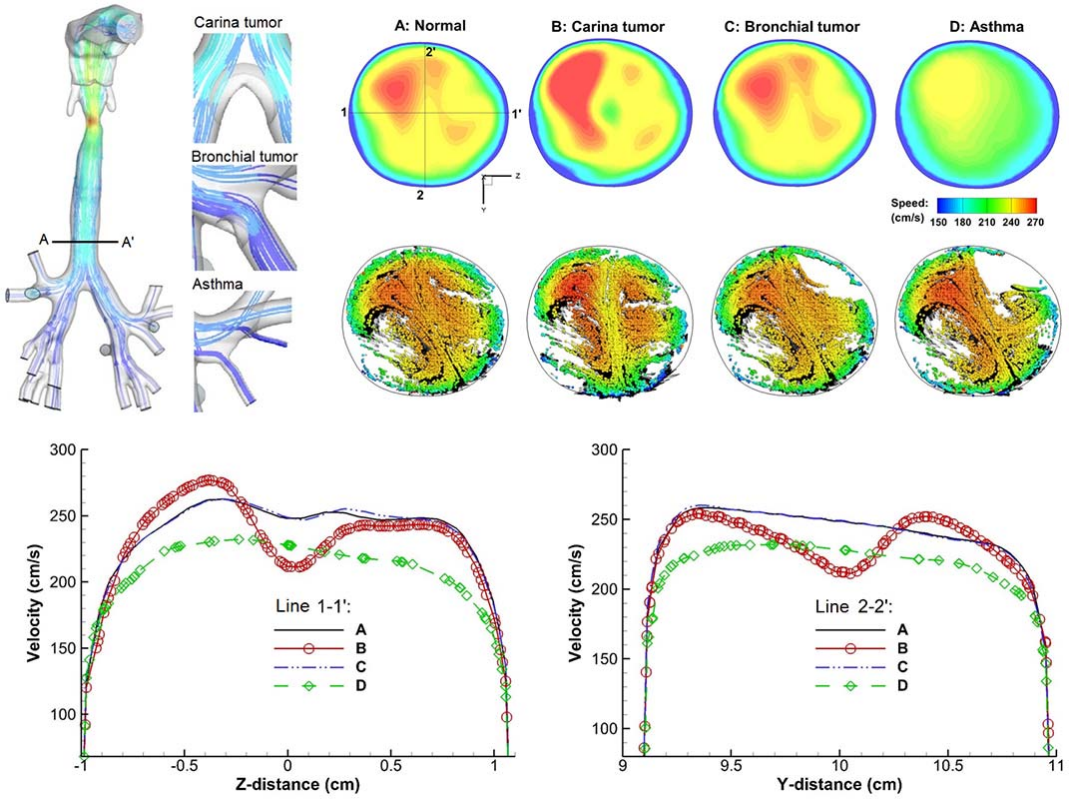
Comparison of expiratory flow fields among the four models of A (Normal), B (carina tumor), C (bronchial tumor), and D (asthma). The presence of an airway obstruction disturbs the exhaled airflow field which will further distort the trajectories of entrained particles and gives rise to different exhaled aerosol profiles. The characteristics of flow distortions depend on the location and size of the airway obstructions.

Figure 2 shows the comparison of expiratory airflows among the four models. The presence of an airway obstruction noticeably alters the airflow field near the diseased site as shown by the distorted streamlines and velocity distributions (top panel in Fig. 2). The variation of the velocity field is further visualized using the cross-sectional particle distributions (middle panel) close to the carina (Slice A–A’). The tracer particles have a diameter of 1 µm and closely follow the airflow. It is observed that both the location and size of the airway obstruction influence the exhaled flows, which give rise to different expiratory particle patterns. The lower panel of Fig. 2 shows the velocity distributions at Slice A–A’ of the four models in both horizontal (Z) and transverse (Y) directions. Compared to the control case (Model A), the most dramatic difference is noted in Model B (carina tumor) which has the largest tumor size and is closest to the sampling plane A–A’. In contrast, Model C (segmental bronchial tumor) gives very similar velocity profiles due to its smaller tumor size and larger distance from Slice A–A’. However, this similar airflow does not necessarily imply similar particle profiles, which depends on both local airflows and upstream particle histories [44]. The time-integrative nature of the particle behaviors can be seen clearly by comparing Model A and C in terms of their similar velocity profiles and different particle distributions at Slice A–A’ (Fig. 2). Lower velocities are observed in Model D (Fig. 2) due to the severely constricted segmental bronchus and associated higher flow resistances. There is a spot that is devoid of particles in the top right corner of Model D, which is presumably caused by the two constricted bronchus. The difference in airflows gradually diminishes as they move towards the mouth; however, the particle profiles are still different due to their time-integrative properties.

### 3.2. 2-D comparison of exhaled aerosol-fingerprints (AFPs)

**Figure 3:**
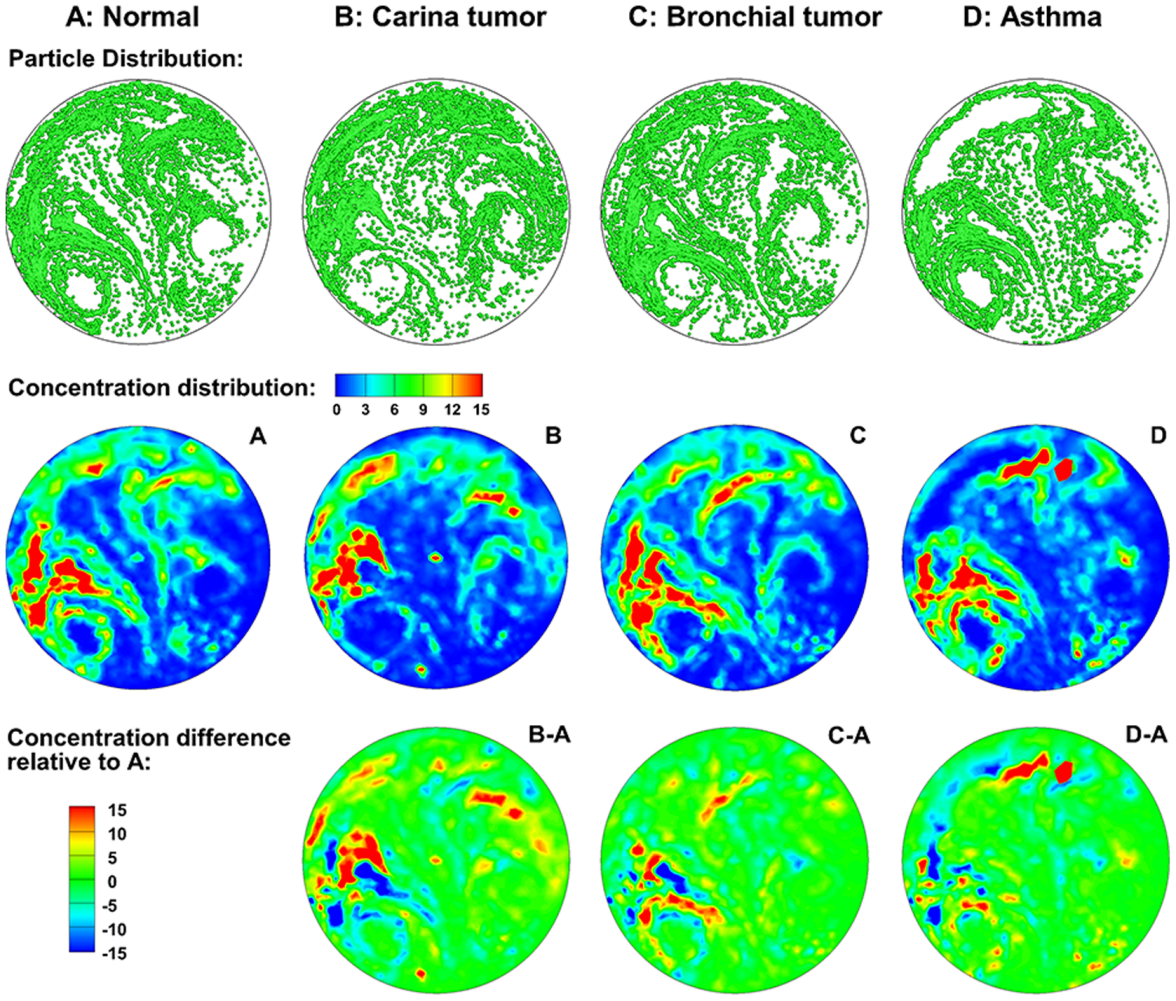
Visual and quantitative comparison of exhaled aerosol fingerprints (AFPs) among the four models. The first row shows particle distributions collected at the mouth. The second row shows the particle concentration distributions, and the third shows the concentration differences relative to the normal condition.

The exhaled particles collect into a pattern that is unique to the lung structure and can be considered the “fingerprint” of that lung. The first row of Fig. 3 displays particle distributions collected at the mouth for an aerosol size of 1 µm and a flow rate of 30 L/min. Overall, each of the four models exhibits a pair of vortexes and an asymmetrical aerosol distribution, the latter of which may stem from the asymmetry of the right and left lungs. However, discrepancies in aerosol distributions are still apparent among the four models. Compared to Model A, Model B (tracheal carina tumor) and C (left segmental bronchial tumor) both exhibit very different patterns. First, the two vortexes and the central stripe in Models B and C are much less defined. The left vortex almost vanishes in Model B. Secondly, for Models B and C with obstructive tumors, an increased portion of aerosols are trapped in the airway due to elevated inertia impaction. However, even though the particle patterns of Models B and C look similar, careful examinations still reveals discernible differences. The presence of a carina tumor (Model B) disturbs the aerosol distribution in both the lower-left and lower-right regions, while the influence from the left segmental bronchial tumor is mainly limited to the lower-left region (top panel in Fig. 3). For Model D with two severely constricted bronchi, the exhaled aerosol profile resembles that of Model A, except for one crescent-shaped region at the upper left corner that is devoid of particles. This observation clearly corroborates the hypothesis that the exhaled aerosol distribution is the fingerprint of the lung structure, which can be used to probe structure remodeling by lung tumors and other respiratory diseases.

Even though the particle distributions look different among the four models, they may not accurately represent the concentration distribution due to particle overlapping. The second row of Fig. 3 shows the relative particle concentrations (i.e., the ratio of local particle concentration to the overall concentration) with red representing high concentrations. For a given model, the particle (first row) and concentration (second row) distributions resemble each other in terms of the overall pattern. However, the concentration image is able to identify the peak particle accumulations (red color), which the particle distribution image is incapable of identifying.

To highlight the variation of AFPs with different model geometries, the relative concentrations compared to the normal condition (baseline) are plotted in the third row of Fig. 3. As such, image A–A (not shown) should have zero concentration everywhere. The other three images (B–A, C–A, D–A) exhibit both positive and negative values, with the red color representing the peak concentration of the abnormal case, while the blue color representing the peak concentration of the baseline case. Therefore, if two adjacent spots have a similar pattern but in opposite colors (red vs blue), the shifting between these two spots can be used to distinguish that disease. Considering the red and blue spots at the top of D–A image, the constricted bronchi in Model D caused the blue spot in the control case to shift toward the top-left (Fig. 3).

### 3.3. Spatial distribution of exhaled aerosol particles

Even though it is effective to differentiate exhaled AFP patterns visually, this process can be slow if there are a large number of images. In order to develop an automated pipeline to quantify exhaled AFP profiles, we explore multiple analytical approaches to distinguish the complexity between different exhaled aerosol profiles. Automated methods that have been tested include spatial scanning, fractal dimension, lacunarity analysis, and multifractal spectra.

**Figure 4:**
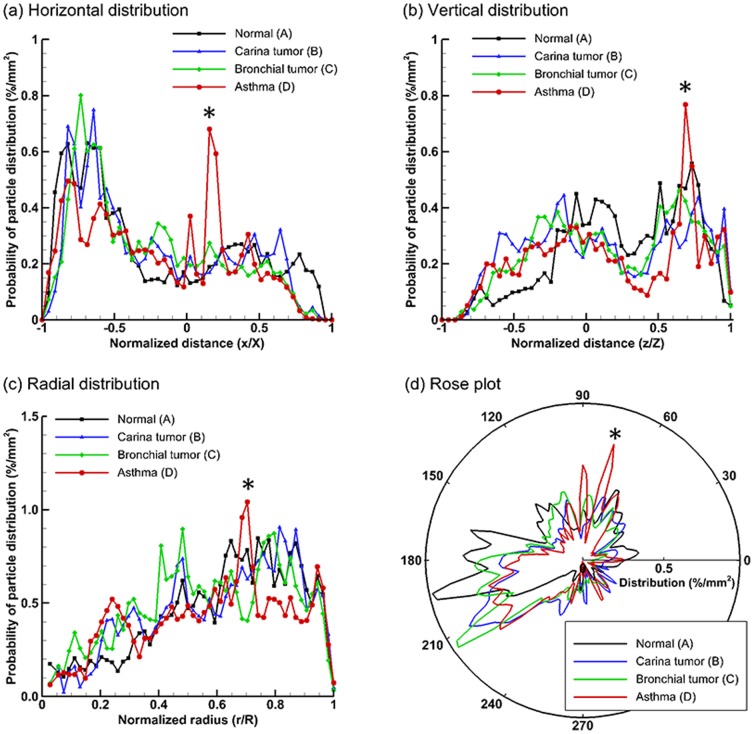
Statistical analysis of exhaled particle distributions at different directions: (a) horizontal, (b) vertical, (c) radial, and (d) circumferential (rose plot). The patterns of exhaled particles among the four models can be distinguished by comparing the spatial distributions of particles in two mutually orthogonal directions.

Figure 4 shows the statistical distributions of exhaled particles in different directions. Taking Fig. 4a as an example, each point hereof represents the probability that the exhaled particles could be found at a specified horizontal distance *x/X*. In this example, the AFP image has been evenly divided into 50 bins along the horizontal direction. The number of particles in each bin is counted and normalized by the total exhaled particle numbers and the area of the bin, yielding the probability of particle distribution (%/mm^2^) at *x/X*. This is equivalent to scanning the AFP image in *x* direction with a scan resolution of *D*/50, with *D* being the diameter of the image. In order to quantify the spatial characteristics of the exhaled particle patterns, the images are scanned in four directions: horizontal, vertical, radial, and circumferential (rose plot). Generally, each airway model considered in this study exhibits a unique profile of spatial distribution probabilities, and therefore is applicable to supplement the classification of airway anomalies. Considering Figs. 4a and 4b, two spikes are observed for Model D (asthma) at *x/X*≈0.2 (Fig. 4a) and *z/Z≈*0.65 (Fig. 4b), which collectively point to the hot spot located at the normalized Cartesian coordinate (0.2, 0.65) as shown in Fig. 3 (concentration distribution D). The same hot spot also manifest itself as a spike in Fig. 4c at *r/R*≈0.7, and in Fig. 4d at θ≈70°. This indicates that directional particle distribution is a sensitive index of spatial pattern which could possibly be quantified with two mutually orthogonal directions.

### 3.4 Fractal, lacunarity, and multifractal analysis

#### 3.4.1 Fractal dimension analysis

**Figure 5:**
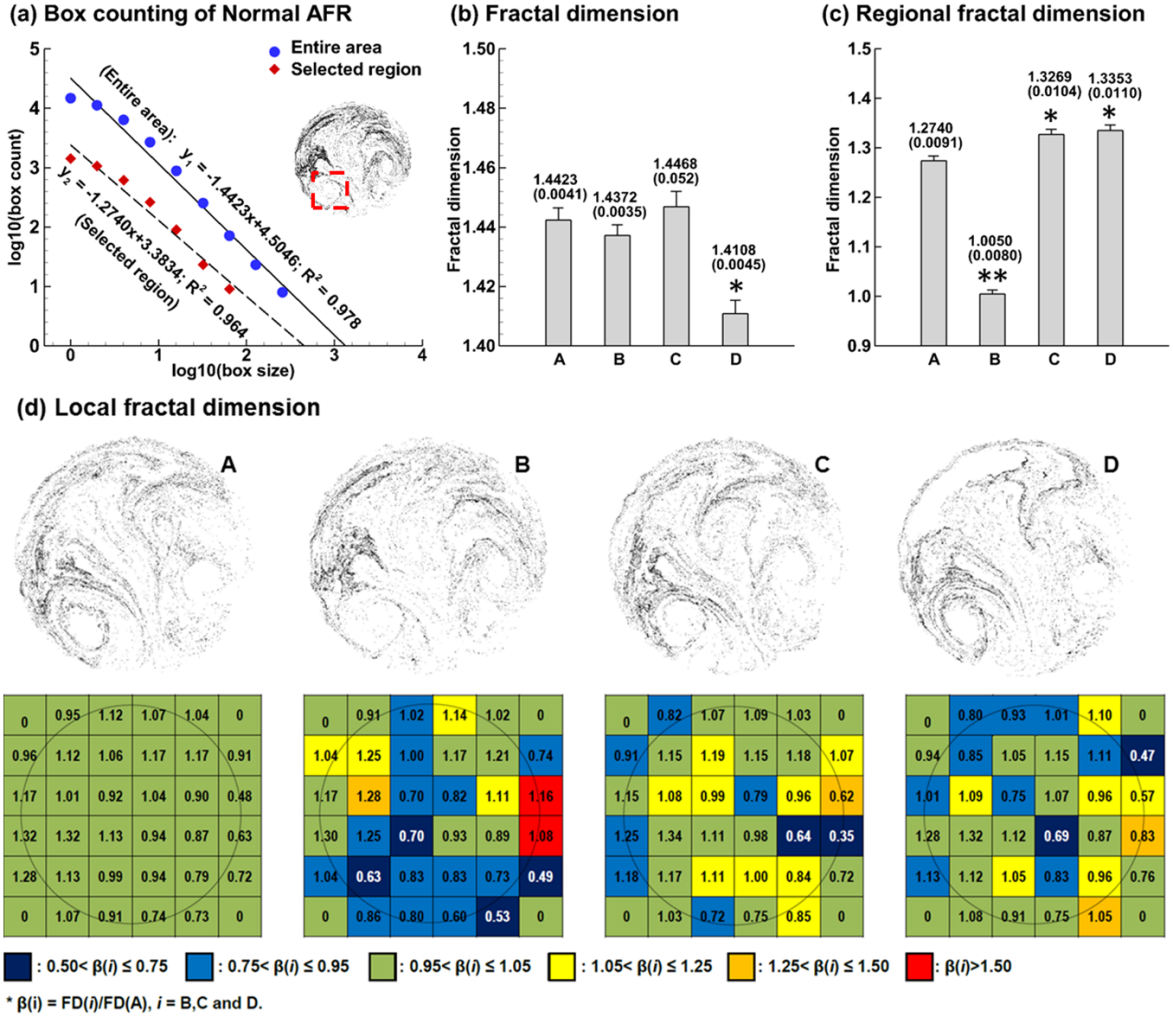
Fractal analysis of exhaled particle distributions using Box Counting method. Calculation of fractal dimension (FD) of Model A using regression analysis is exemplified in (**a**). FDs FDs (±SD, n = 5) for the four models are shown in (**b**) and (**c**) for the entire image and selected region of interest (ROI), respectively. Significance indicated by *(p<0.05) and **(p<0.01). (**d**) shows the local FD distribution on a normalized caliber size of 1/6×1/6. The color code was based on the fractal dimension ratio β(i) = FD(*i*)/FD(A), *i* = B, C and D. The color pattern is unique to each airway abnormality.

Monofractal analysis of exhaled aerosols using the box counting method is shown in Fig. 5 for the four models. We consider the fractal dimensions from two perspectives: in the entire sample image and in the selected region of interest (ROI), as illustrated in Fig. 5a. The correlation factor of data linear regression is 0.978 for the entire region, indicating that the particle distribution exhibits a statistically fractal feature (Fig. 5a). The local distribution also exhibits a fractal feature (*R^2^* = 0.964), except that it has a smaller fractal dimension (FD_ROI_ = 1.274) and is less complex than that of the entire region (FD_Entire_ = 1.4423).

Comparison of FD based on the entire region among the four models is shown in Fig. 5b. The FD standard deviation for each model has been calculated from five test cases with stochastically generated inlet particle profiles (n = 5). Significance is indicated by *(p<0.05) and **(p<0.01). The deviation of FD from the normal case is consistent with the level of airway remodeling even though small in magnitude. Model C (small bronchial tumor) and Model B (large tumor) cause insignificant variation in FD while Model D (asthma) causes a larger FD variation (p<0.05). The lower FD (1.4108) of Model D versus the control case (1.4423) corroborates the prior report that asthmatic lungs have decreased FD values compared to non-asthma controls [45]. In part, this decrease might be explained by the ventilation loss due to airway constrictions.

In contrast to the small variations of entire-region-based FDs, the variation of local FDs is more pronounced. For the region of interest (ROI) (red square in Fig. 5a), the local FD of Model B is significantly lower than the control (p<0.01). In view of the proximity of FD values for Model A, C, and D, it is possible that this selected ROI is largely affected by the carina tumor and not by the bronchial tumor (B) or asthmatic bronchi (D). Local FD distribution on a 6×6 grid is displayed in Fig. 5d. For each grid, the color code is based on the FD ratio β(i) = FD(*i*)/FD(A), *i* = B, C and D. Again, the color patterns for the four models are different from each other, and are unique to each airway abnormality.

#### 3.4.2 Lacunarity analysis

**Figure 6:**
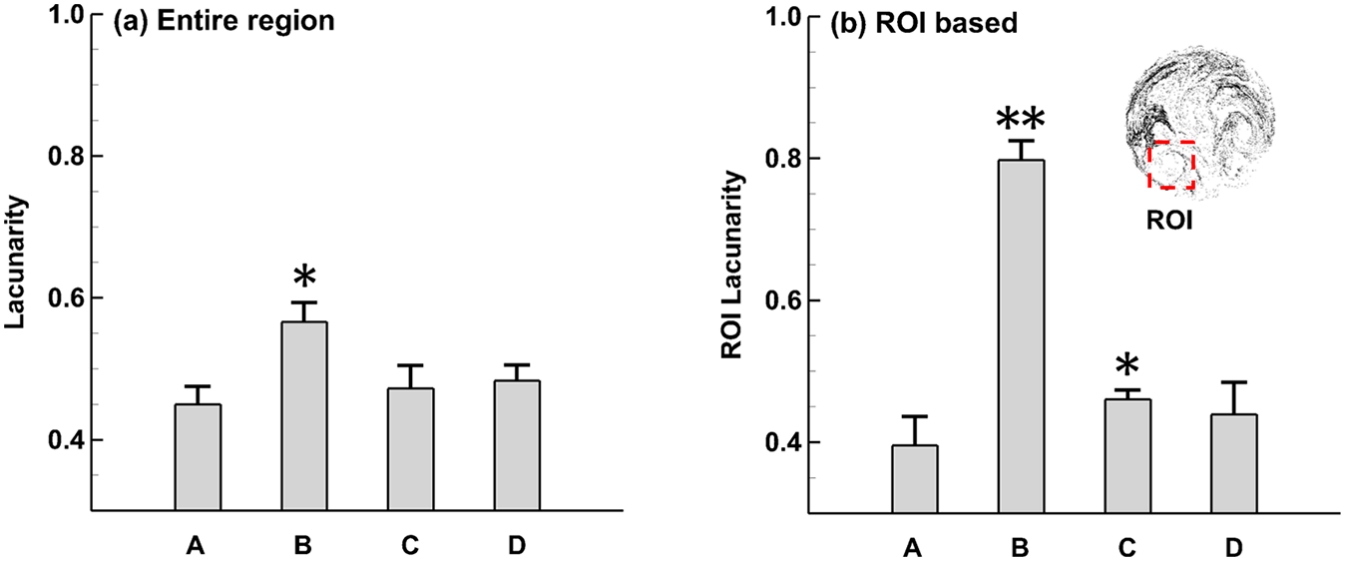
Comparison of lacunarity values FDs (±SD, n = 5) among the four models for (a) entire region, and (b) selected region of interest (ROI). Significance indicated by *(p<0.05) and **(p<0.01).

In general, measures of lacunarity correspond to visual impressions of uniformity, where low lacunarity implies homogeneity and high lacunarity implies heterogeneity. From Fig. 6a, the differences of lacunarity among the four models are more pronounced than those of fractal dimensions for both the entire sample and selected region of interest (ROI). Specifically, the lacunarity of Model B differs significantly (P<0.01) from the control case (Model A) even though their fractal dimensions are similar. As discussed before, fractal dimension and lacunarity are statistical indexes of complexity and heterogeneity, respectively, and do not necessarily correlate to each other. Knowing lacunarity helps to separate exhaled aerosol images with close fractal dimensions. Comparing ROI-based images in Fig. 6b, Model B (carinar tumor) has the largest lacunarity (Fig. 6b) and the smallest fractal dimension (Fig. 5b) among the four models while the variations among the other three models are insignificant. This suggests a strong correlation between the carina tumor to large variations of fractal dimension and lacunarity in the ROI, which will be further analyzed using multifractal spectrum analysis in the following section.

#### 3.4.3 Multifractal analysis

**Figure 7:**
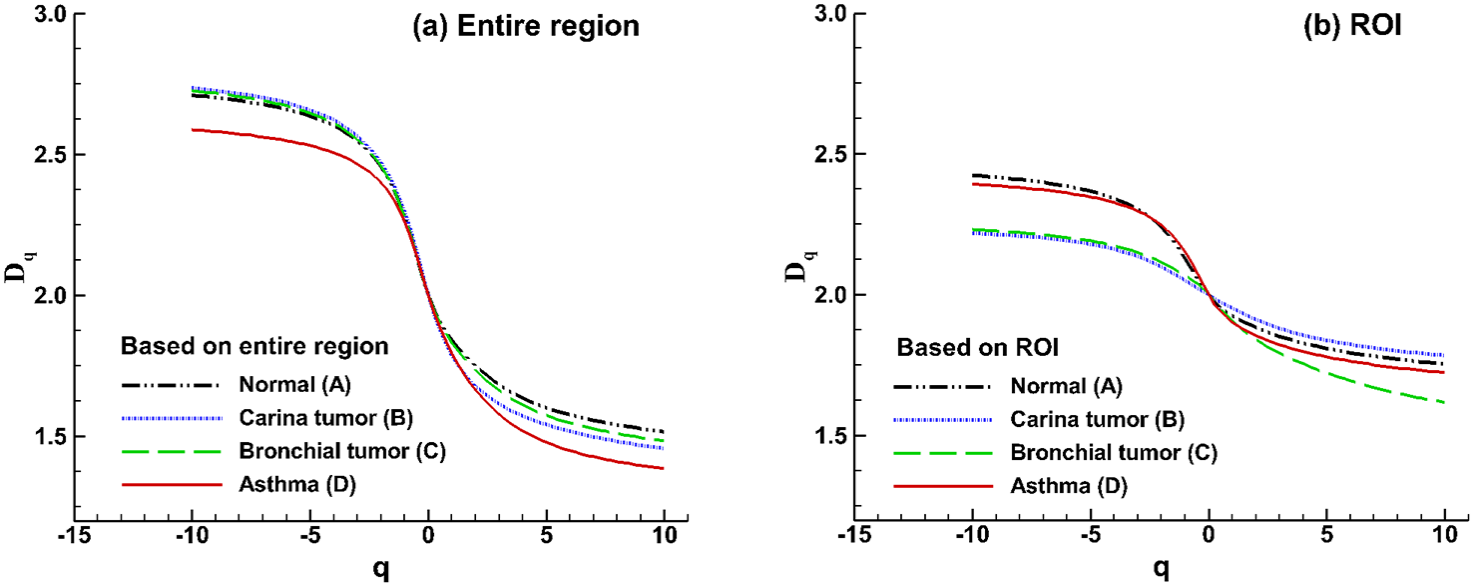
Spectra of generalized dimensions *Dq* versus *q* among the four models for (a) the entire region and (b) the selected region of interest (ROI).

Multifractal patterns are intrinsically more complex than monofractals. The visually patchiness of exhaled aerosol profiles suggests that different scaling properties may exist. Figure 7 shows the spectra of generalized dimension *D_q_* versus the scaling exponent *q* for both the entire region and selected ROI. In all cases, *D_q_* is a monotonically decreasing function of *q*, indicating that the exhaled aerosol patterns exhibit multifractal features [46] and are more appropriately described by the multifractal spectra rather than by the box-counting fractal dimensions alone. In the case of a monofractal, the *D_q_* spectrum should be a constant line, which is not observed in Fig. 7. Moreover, the *D_q_* spectra of the ROI cases are flatter than those of the entire region, suggesting that local patterns are more like monofractals (even though they are not), while the overall patterns are more like multifractals.

**Figure 8:**
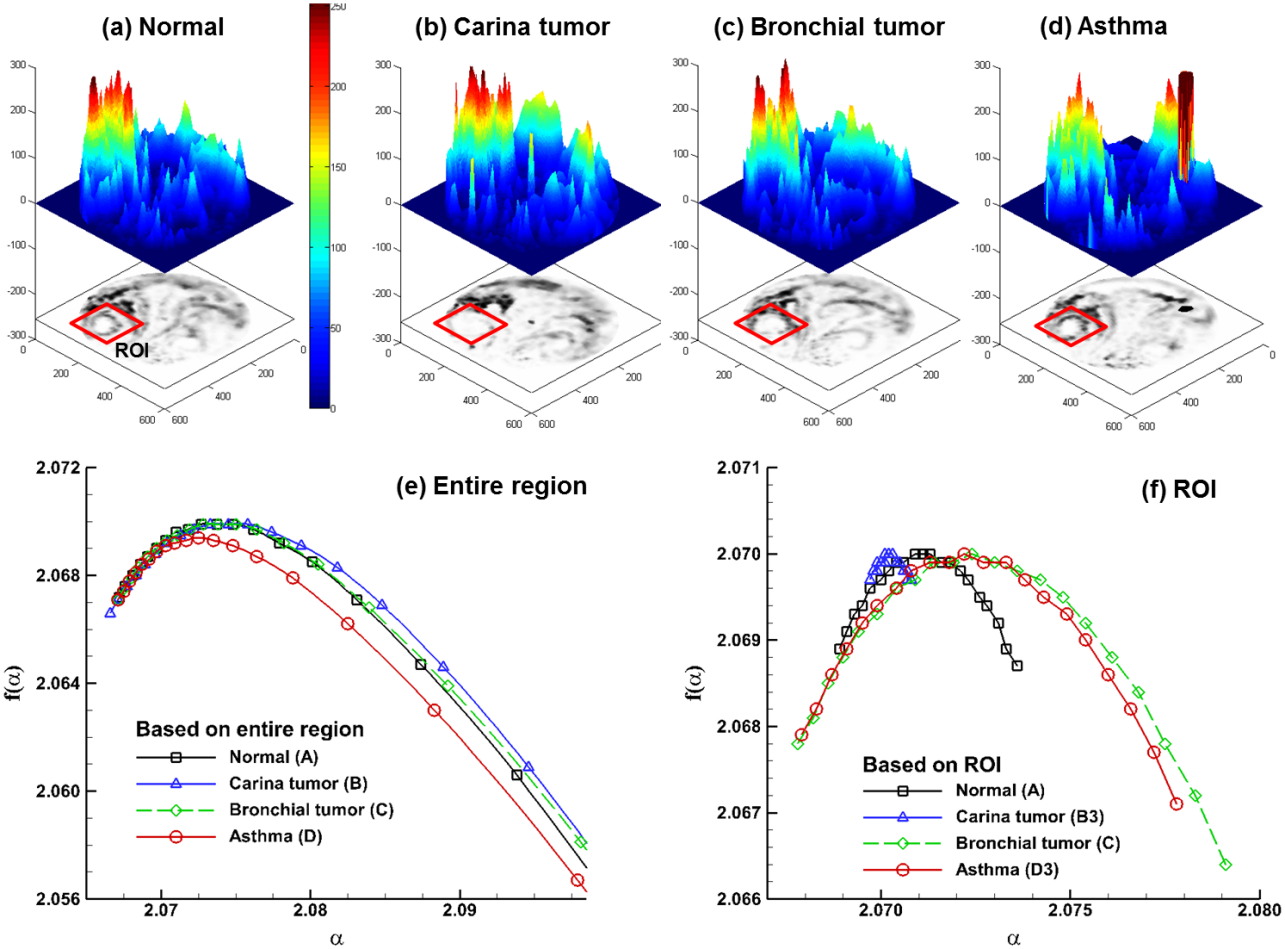
Multifractal analysis of exhaled particle concentrations. The 3-D plots of particle concentrations are shown in (**a**), (**b**), (**c**), (**d**). Comparison of the multifractal spectra among the four models are shown in (**e**) for the entire region and in (**f**) for the selected region of interest (ROI).

The multifractal spectra for the gray-sale images of the exhaled aerosol concentration profiles are shown in Fig. 8. The aerosol concentration images are first shown as the 3-D plots (Figs. 8a–d), which exhibit very different patterns among the four models. Considering the entire-region analysis (Fig. 8e), a small geometric deviation such as bronchial tumor (Model C) leads to a similar profile as that of the control case, while large geometric variations leads to spectra profiles that are much different from the control, which is consistent with Fig. 5. For the selected ROI, the spectra are more symmetrical than those of the entire region. The ROI-based spectra also have a smaller range of *f(α)* as well as a narrower range of *α* compared to those of the entire image suggesting lower multifractality of the ROI images. Particularly, the ROI-based spectrum for Model B has the smallest ranges of *f(α)* and *α* (Fig. 8f), which also has the smallest monofractal dimension (Fig. 5c) and largest lacunarity (Fig. 6b). This is in line with results in previous studies [20], [47] that a pattern with a more asymmetric spectrum and a narrower range of α generally has higher density and lower lacunarity. Examples of such patterns include soils with massive structures and low porosity [47] and vascular beds with high complexity and lower emptiness [20]. In this study, the exhaled aerosol profiles of the entire region are more complex and heterogeneous than that of the ROI. Apparent differences in the ROI spectra are also observed among the four models (Fig. 8f), lending further evidence that multifractal analysis might be adequate in identifying the geometry-associated aerosol variations.

### 3.5 AFPs for asthma with varying severity

**Figure 9:**
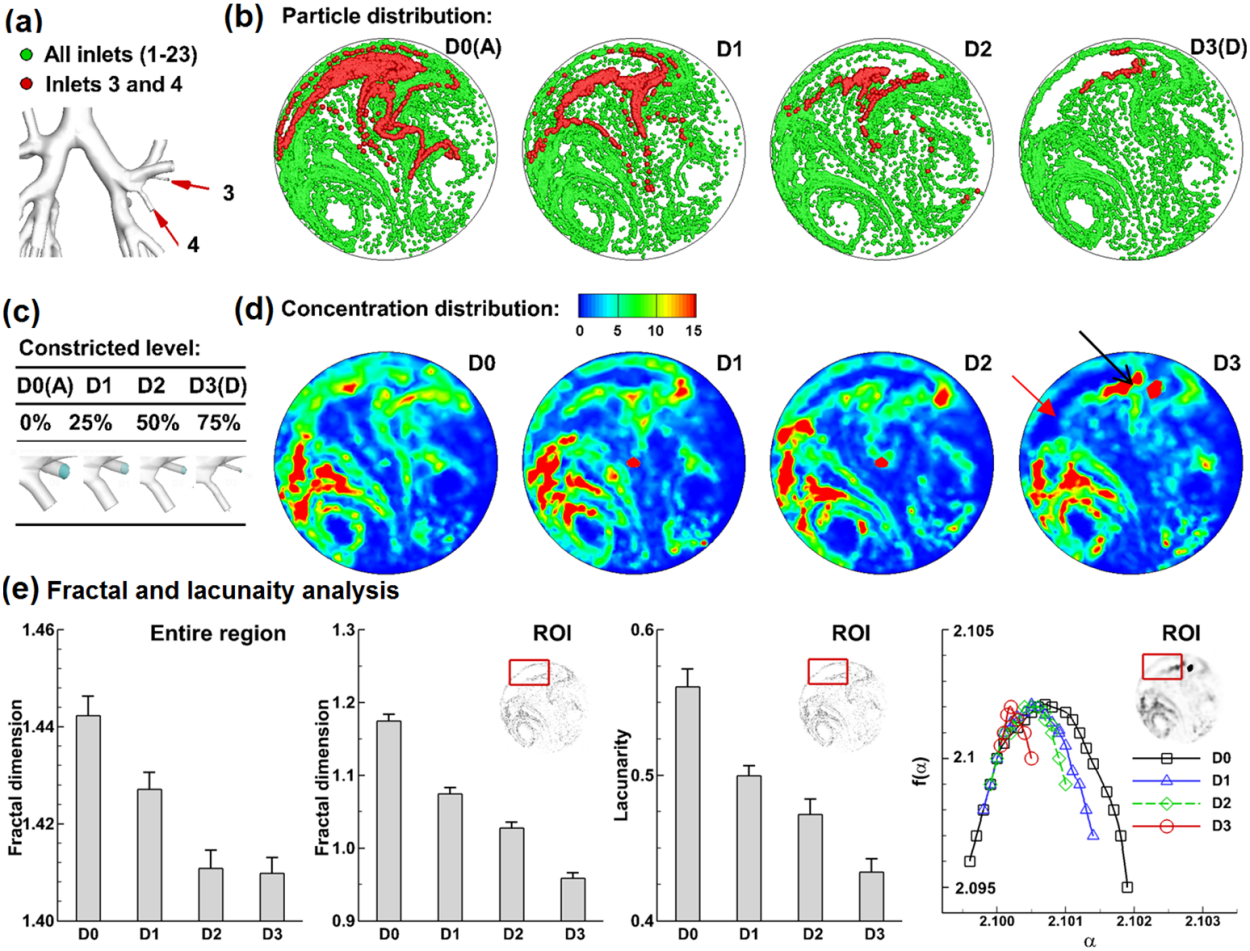
Exhaled aerosol fingerprints (AFPs) for asthma with increasing severity. (**a**): constricted segmental bronchi 3 and 4 with increasing severities. The constriction levels are shown in (**c**). The exhaled particle distribution is shown in (**b**) while the concentration distribution is shown in (**d**). Fractal analysis FDs (±SD, n = 5) for the entire image and selected ROI is shown in (**e**) in terms of fractal dimension, lacunarity, and multigractal spectra.

To test whether the exhaled AFPs are sensitive enough to distinguish the pathologic states of respiratory diseases, four levels of airway constrictions (D0, D1, D2, D3) caused by asthma have been considered, as illustrated in Figs. 9a and 9c. Exhaled particle distributions are shown in Fig. 9b. It is noted that the crescent-shaped void at the upper-left corner becomes more obvious with increasing severities. To further test the sensitivity of the aerosol voids to the disease severity, particles are released only from the ROI and their exhaled locations are plotted in red (Fig. 9A). For the zero-level constriction (D0), red particles are observed enclosing the region that is otherwise aerosol-void for asthmatic scenarios (D1–3). With increasing severity, red particle contours shrink progressively in space (Fig. 9B), with drastically elevated concentration in certain regions (solid arrow) and decreased concentration in other regions (hollow arrow) (Fig. 9D), reflecting the asthma condition at the ROI. As a result, these tagged particles could not only be used to evaluate the severity of airway constriction, but also to discover the location of the disease.

Fractal analyses of exhaled AFPs with asthma of varying severities are shown in Fig. 9E. Again, the standard deviation for each case has been calculated from five tests with different stochastically generated inlet particle profiles. For both the entire region and selected ROI (upper left), there is a progressive decline in FD for airways with increasing severities. Concerning the ROI, the FD and lacunarity of each asthma case (D1−3) are different from the control D0. In light of the multifractal spectra of the ROI, increasing airway constriction leads to continuous narrowing of both *α* and *f(α)*, which clearly distinguishes the four asthma states considered in this study.

## Discussion

The use of multiple analytical techniques is becoming increasingly pertinent when exploring complex biological systems. In this study, we demonstrated the feasibility of a coupled CFD-fractal approach to quantitatively distinguish the exhaled AFP patterns from healthy and diseased lung models. Physiology-based numerical modeling has been employed to predict the exhaled aerosol patterns (fingerprints), which revealed notable variations of exhaled aerosol fingerprints among the four models in both visual patterns and fractal measures. Compared to our previous study [13] that was limited to visual patterns and a qualitative manner only, the current study quantified the exhaled AFP patterns by exploring multiple analytical approaches, including concentration disparity, spatial scanning, monofractal, lacunarity, and multifractal analysis. These approaches collectively generated a feature vector of the AFP pattern, which could be further used for automated classification of the AFPs and diseases. The concentration disparity provides a more informative comparison than the particle distribution presented in the previous study [13]. The spatial scanning can quantify particle distributions in either (*x, y*) or (*r,* 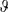) directions and is a sensitive index of the spatial pattern. In light of the fractal analysis, the ROI-based fractal dimension and lacunarity can be significantly correlated with the severity of airway obstruction. In addition, multifractality can identify the subtle differences in the exhaled aerosol profiles. The capacity to differentiate not only gross differences but also subtleties among aerosol fingerprints is highly desirable. It provides a useful tool in decoding the complexity of such fingerprints and thus can be used to monitor the pathogenesis of an airway disease or track the therapeutic outcomes of an intervention protocol. Besides, one particular advantage of the physiology-based modeling is that the results will not be confounded by any other factors except the factor of interest.

The fractal dimensions (FD) of exhaled AFPs are observed to decrease with increasing disease severities. A decrease in FD indicates a loss in complexity, which reflects a decrease in space-filling ability of diseased airways. Airway remodeling is a consequence of chronic injury and repair, whose site and severity can vary significantly. The structural variations considered in this study (Table 1) are small and represent a conservative evaluation of the performance of the proposed AFP-based breath test. For example, airway constrictions in some asthma patients are much more severe than the model D in this study. Fatal asthma can have 44% closure of the whole airway [45]. In this sense, a more pronounced variation of fractal measures is expected in clinical practices, which should be even more useful for diagnostic purposes.

The AFP-based breath test is envisioned to be similar to a personal air sampling system. The patient inhales tracer particles and then exhales. The exhaled particles are collected on a fibrous or pored membrane filter [48] for a prescribed sampling period. To minimize artifacts due to variations in breathing or body posture, the breath rate and body posture should be standardized during the test. Exhaled profiles or fingerprint patterns can be quantified with different approaches to distinguish normal versus diseased lungs. This can be achieved via direct image processing, microscope-based counting, fluorescent intensity measurement, or chemical quantification [49]. Particle counting with a microscopy has been used to determine total and local depositions of aerosols in a bifurcating geometry [50]. The concentration of fluorescent tracers can be measured with a fluorometer [51]. The other alternative is to use chemical sensitive tracer particles which change colors upon contacting the filter and generate a pattern that is specific to the respiratory structure of concern [52]. Ideally, tracer aerosols for the breath test should be non-invasive, sensitive, easy to analyze, disease-specific, and repeatable. Results of this pilot study suggest that the first three criteria are attainable.

The eventual breath test will consist of two steps to detect and localize the disease: (1) extraction of image features and (2) classification between images and diseases. The methodology presented in this study has focused on feature extraction only, which will accurately and compactly quantify the image. However, by itself it is not enough to identify or trace back to the disease site. This requires a database of image-diseases and a classification method that correlates the images with their respective diseases. The extracted feature vector will be used as the input to train the clarification function *f(x)* that correlates the image and diseases. Among the many options of classification methods such as neural network, machine vision, and support vector machine (SVM), the SVM algorithm will be selected for future classification studies due to its accuracy and easy-to-use features [53]. To this aim, the open-source software package the Library for Support Vector Machines (LIBSVM) could be adopted for data classification [54].

As a proof-of-concept study, we employed ideal breathing conditions to assess the feasibility of the proposed breath test, e.g.: same flow velocities for both normal and pathological lung models. It is noted that a patient with respiratory distress may breathes differently, which can alter the exhaled AFP patterns. To accurately detect an airway abnormality, it is necessary that the pattern of exhaled AFP persists over a certain range of breathing conditions even though its pattern details may vary. Practically, the respiration bias can be minimized by instructing the patient to inhale steadily and by activating the exhalation sampling only when the patient breathes within the acceptable range. Future studies should also be conducted to determine the sensitivity of the AFP-based breath test under various breathing conditions. Quantifying the respiration effect will help to determine the detection sensitivity, the tolerance of breathing deviations, and the optimal breathing maneuvers for the breath test [55].

Other limitations of this study include the assumption of steady flows, no humidity, no charge effect, rigid airway walls, and small sample size. Previous studies have highlighted the significance of transient breathing [56], hygroscopic growth [57], [58], particle charge effects [59], [60], dynamic glottis [61], and intersubjective variability [62], [63]. Generally, structure variations are also accompanied by tissue property and functional changes, which are expected to result in a larger magnitude of fractal changes. Concerning the sample size, the geometry models considered are from limited subjects and do not account for intersubject variability. Each of these factors affects the realism of the model predictions in relation to actual performance of the aerosol breath test. These limitations should be addressed in order to develop more physically realistic models. Future numerical studies with more realistic models and a larger sample size, as well as complementary *in vitro* tests, are necessary to advance our knowledge of the feasibility and efficiency of this new lung diagnosis protocol.
